# Comparative analysis of dental-derived stem cells and alternative stem cell sources: properties and regenerative functions

**DOI:** 10.3389/fbioe.2025.1717849

**Published:** 2025-11-18

**Authors:** Kexin Yang, Shengmeng Yuan, Yuhao Wang, An Lin, Fangjun Huo, Zhaorui Jin, Chao Yang, Weidong Tian

**Affiliations:** 1 Department of Oral and Maxillofacial Surgery, West China Hospital of Stomatology, Sichuan university Chengdu, Chengdu, Sichuan, China; 2 State Key Laboratory of Oral Diseases and National Clinical Research Center for Oral Diseases and Engineering Research Center of Oral Translational Medicine, Ministry of Education and National Engineering Laboratory for Oral Regenerative Medicine, West China Hospital of Stomatology, Sichuan University, Chengdu, Sichuan, China; 3 Chengdu Shiliankangjian Biotechnology Co., Ltd., Chengdu, Sichuan, China

**Keywords:** mesenchymal stem cells, dental stem cells, cell therapy, tissue regeneration, regenerative medicine

## Abstract

Due to their accessibility, wide range of sources and unique biological characteristics, dental stem cells have broad application prospects in regenerative medicine. This cell population mainly includes dental pulp stem cells, periodontal ligament stem cells, stem cells from deciduous teeth, and dental follicle stem cells. In addition, dental stem cells have good microenvironment-specific immunomodulatory functions, including inhibiting T cell activation, promoting the polarization of regulatory T cells and regulating the phenotype of macrophages, thereby promoting tissue repair and reducing inflammation. These advantages are complemented by its strong osteogenic differentiation ability, providing a new strategy for oral tissue regeneration, and providing broad prospects for the treatment of nervous system related diseases due to its ectodermal homology with neural crest. This review systematically summarizes the major advantages of dental stem cells in the field of regenerative medicine, outlines current progress in clinical translation, and discusses future research directions, while critically comparing their therapeutic potential and challenges with other mesenchymal stem cells sources to guide seed cell selection and clinical applications.

## 1 Introduction

**FIGURE 1:**
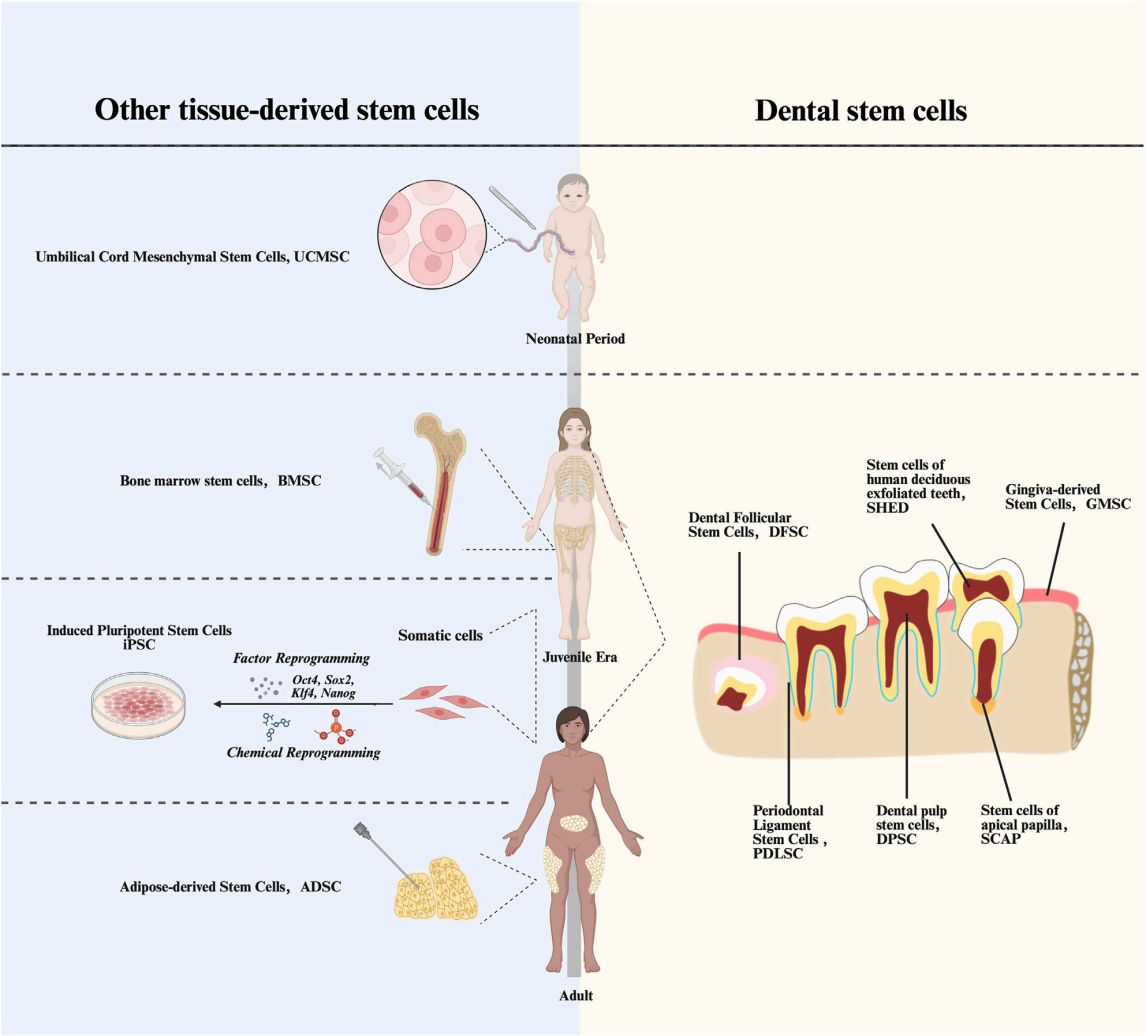
Timeline of acquisition of various types of MScs. UCMSCs, Umbilical Cord Mesenchymal Stem Cells; BMSCs, Bone Marrow Stem Cells; iPSCs, Induced pluripotent stem cells; ADSCs, Adipose-derived Stem Cells; DPSCs, Dental Pulp Stem Cells; PDLSCs, Periodontal Ligament stem Cells; DFSCs, Dental Follicle Stem Cells; SCAPs, Stem Cells from Apical Papilla; GMSCs, Gingiva-derived Mesenchymal Stem Cells; SCAPs, Stem Cells from Apical Papilla.

Mesenchymal stem cells (MSCs) are adult stem cells that are widely distributed in various organs of vertebrates, such as adipose, teeth, bone marrow, and umbilical cord and placenta of newborns (Zhao et al., 2025) (Figure 1). Due to their superior self-renewal, differentiation potential, immunomodulation and other abilities, they are now regarded as an important tool for various types of tissue regeneration and treatment of immune and inflammatory diseases (Peishan et al., 2023).

As the most extensively studied MSCs, bone marrow mesenchymal stem cells (BMSCs) demonstrate remarkable differentiation potential, showing therapeutic promise for osteoporosis and bone defects (Song et al., 2025). However, their clinical application is constrained by the invasive bone marrow aspiration procedure required for isolation, which may cause secondary trauma to patients (Rossi et al., 2023). While adipose-derived stem cells (ADSCs) offer wider tissue accessibility, their weak chondrogenic differentiation ability limits their application in bone tissue engineering (Lee et al., 2012). The immunomodulatory effects of Umbilical cord mesenchymal stem cells (UCMSCs) diminish, with their aging *in vivo*, which may affect their clinical applications (Paladino et al., 2017).

In contrast, dental stem cells (DSCs) can address these limitations while exhibiting unique immunological and sourcing advantages. Their low immunogenicity enhances allogeneic transplantation efficacy, and their availability from clinical waste materials (e.g., exfoliated deciduous teeth and extracted wisdom teeth) eliminates invasive procedures and ethical concerns (Pires et al., 2025). DSCs primarily include dental pulp stem cells (DPSCs), stem cells from human exfoliated deciduous teeth (SHEDs), periodontal ligament stem cells (PDLSCs), and dental follicle stem cells (DFSCs) (Umapathy et al., 2025). These ectoderm-derived stem cells not only exhibit regenerative potential in oral tissue regeneration, but also show promising preclinical results that may indicate advantages for nerve regeneration compared to other MSCs (Gopalarethinam et al., 2023). These characteristics endow dental stem cells with broader prospects for therapeutic applications.

Through systematic comparison of DSCs with other stem cell sources in terms of accessibility and biological properties, coupled with an in-depth exploration of their therapeutic potential across multiple pathological conditions, this review aims to provide valuable insights for future research and clinical translation.

## 2 Methodology of literature review

This review was designed as a methods-informed narrative review to provide a transparent overview of DSCs and their comparison with MSCs. Relevant studies were retrieved from PubMed/MEDLINE, Embase, Web of Science, and Scopus, covering the period January 2000–September 2025. Keywords combined terms related to DSC subtypes (DPSCs, SHEDs, PDLSCs, SCAPs, DFSCs, GMSCs) and their biological functions (immunomodulation, osteogenesis, neurogenesis, regeneration, clinical trials) using Boolean operators.

We included peer-reviewed English-language original studies (*in vitro*, animal, or early-phase clinical) that characterized DSC identity, differentiation, or immunomodulatory properties, or directly compared DSCs with other MSCs. Editorials, conference abstracts, and studies lacking primary data were excluded.

Two authors independently screened titles and abstracts, followed by full-text assessment. Disagreements were resolved by discussion. The final evidence was grouped thematically—cell identity, immunomodulation, osteogenic/neurogenic differentiation, and clinical applications—and summarized qualitatively, highlighting comparative findings and limitations of current data.

## 3 Types and properties of dental stem cells

Originating from the ectomesenchyme, DSCs are a class of mesenchymal-stem-cell-like with self-renewal ability and multi-lineage differentiation potential. DSCs share not only the common of features and expression markers, but also their unique characteristics.

### 3.1 DPSCs

Dental pulp tissue is differentiated from the dental papilla, which performs functions such as repair, nutrition, and dentin formation within the dental tissues. Gronthos et al. first isolated DPSCs from pulp tissues of third molar obstruction in 2000 and described their basic properties (Gronthos et al., 2000). The application of DPSCs in tooth restoration and regeneration has been widely studied. Some scholars injected DPSCs into a small porcine periodontitis model and showed that they have a lower cellular senescence rate, higher proliferation rate, and stronger osteogenic maintenance ability, and can promote periodontal regeneration (Ma et al., 2019). In addition to this, DPSCs have lower immunogenicity, low levels of major histocompatibility complex class I (MHC-I) expression, and negative MHC class II expression (MHC-II) (Uccelli et al., 2007). Furthermore, several studies have shown that DPSCs retain their stem cell properties after cryopreservation (Wang et al., 2024), DPSCs culture can be established from extracted human molar teeth with high efficiency even when whole teeth are cryopreserved for up to a month (Perry et al., 2008), demonstrating the potential of DPSCs for facile preservation and cell banking. These advantages have facilitated the rapid clinical translation of DPSCs. A recent multicenter randomized clinical trial conducted in China demonstrated that human DPSCs injection exhibited a favorable safety profile and significantly improved clinical outcomes in the treatment of stage III periodontitis (Liu et al., 2025). This milestone study marks a pivotal step forward in the clinical application of DPSCs.

### 3.2 PDLSCs

The periodontal membrane is derived from the dental follicle of the dental embryo, which is an important structure connecting the alveolar bone to the dentin and helps protect the health of the teeth and alveolar bone by evenly distributing the pressure on the teeth during mastication (Seo et al., 2004). The periodontium undergoes lifelong regeneration and remodeling. It harbors periodontal stem cells expressing MSCs markers STRO-1 and CD146/MUC18 (Wang et al., 2022). These cells are capable of regenerating functional periodontal complexes comprising both ligamentous and osseous structures (Inchingolo et al., 2024). Liu et al. demonstrated successful periodontal defect repair and alveolar bone regeneration through autologous PDLSCs transplantation in a miniature swine model (Liu et al., 2008). A clinical study conducted in Japan demonstrated that autologous PDLSC sheets were effective in treating chronic periodontitis, as evidenced by reductions in probing depth, improvements in clinical attachment levels, and increases in radiographic bone height, with confirmed safety and stability over mid-to long-term follow-up (Iwata et al., 2018). Meanwhile, PDLSCs exhibit neurogenic differentiation potential (Mohebichamkhorami et al., 2022), generating neuronal precursors with morphological and phenotypic characteristics of neurons, astrocytes, and oligodendrocytes, suggesting therapeutic applicability for neurological disorders.

### 3.3 DFSCs

DFSCs are the only type of dental stem cell derived from the dental follicle of the developing tooth germ. Distinct from other types originating from mature dental tissues (e.g., PDLSCs, DPSCs, SHED), their earlier developmental origin confers greater differentiation capacity and broader application potential. These cells exhibit accelerated proliferation under inflammatory conditions and can effectively regenerate functional periodontal complexes in various animal defect models, recapitulating the native sandwich-like multilayered architecture of periodontal tissues (Chen et al., 2025a). Experimental evidence indicates that DFSCs significantly upregulate periostin expression under inflammatory conditions and exhibit superior periodontal tissue regenerative capacity compared to PDLSCs in beagle dog defect repair models (Guo et al., 2017). Furthermore, DFSCs possess particularly remarkable osteogenic potential, which effectively enhances alveolar bone regeneration efficiency (Morsczeck et al., 2023). According to publicly available information, the Center for Drug Evaluation (CDE) of the National Medical Products Administration of China has recently approved a human DFSCs injection developed in Chengdu for clinical trials targeting periodontitis treatment, marking a significant step toward the clinical application of DFSCs. In addition, owing to their neural crest origin, DFSCs have recently attracted attention for their potential in spinal cord injury therapy (Wen et al., 2024). Moreover, studies have demonstrated their capacity to differentiate into retinal precursors and neurons (Lee et al., 2025), suggesting possible applications in the treatment of diverse neurological disorders.

### 3.4 SHEDs

SHEDs are isolated from physiologically shed primary teeth. *In vitro* analyses demonstrate that beyond the conventional trilineage differentiation potential (osteogenic, adipogenic, and chondrogenic) (Miura et al., 2003), SHEDs additionally possess neurogenic and myogenic differentiation capacities (Zeng et al., 2020), suggesting their potential applicability in the treatment of cartilage-related diseases and muscle tissue injuries. SHEDs exhibit superior proliferative kinetics compared to DPSCs, characterized by accelerated population doublings, spontaneous spheroid formation, and upregulated endogenous bone morphogenic protein-2 (BMP-2) expression that enhances osteogenic differentiation and matrix mineralization capacity (Kunimatsu et al., 2018). SHEDs mediate osteogenesis through paracrine mechanisms rather than direct differentiation, establishing osteoinductive niches that recruit murine host osteoprogenitors for *de novo* bone formation. Xenotransplantation studies in immunodeficient murine models reveal SHED’s capacity to generate ectopic dentinoid structures, though complete dentin-pulp complex regeneration remains unachieved (Mohd et al., 2023). SHEDs and DFSCs have similar ability to regenerate periodontal tissue *in vivo*, but SHEDs is more conducive to vascularization and neuralization (Yang et al., 2019). Meanwhile, SHEDs have also been investigated in the treatment of Parkinson’s disease (Chen et al., 2024), corneal repair (Sato et al., 2025) and other conditions, and are increasingly recognized as important seed cells for therapeutic applications in nervous system disorders (Mi et al., 2023), as well as skin and cardiovascular injuries (Qin et al., 2024).

### 3.5 Gingiva-derived mesenchymal stem cells (GMSCs)

Zhang et al. first isolated gingival MSCs from discarded gingival tissues in good health, which showed clonogenicity, self-renewal ability and multiple differentiation capabilities. Lineage tracing studies by Xu et al. revealed dual embryonic origins of GMSCs: 90% originating from neural crest derivatives and 10% from mesodermal precursors. Neural crest-originating GMSCs exhibit enhanced neurogenic differentiation capacity and superior immunomodulatory properties compared to their mesoderm-derived counterparts (Zhang et al., 2009). More surprisingly, unlike BMSCs that exhibit age-related functional decline in migratory capacity and anti-inflammatory responses, human GMSCs maintain phenotypic stability and immunomodulatory competence through extended *in vitro* passaging. In addition, longitudinal culture analyses confirm GMSCs preserve genomic integrity through maintained telomerase activity and stable karyotypic profiles, with no observed tumorigenic potential (Bustos et al., 2014). A previous study obtained GMSCs from 13 to 80-year-old sources for assay, respectively, and found that the expression of cell surface markers did not change with increasing donor age and number of passages. Moreover, GMSCs derived from donors across this age spectrum were found to effectively attenuate inflammatory conditions and contribute to the regeneration of injured lung tissue (Dave et al., 2022). In addition, *In vitro* tumor suppression assays revealed GMSCs significantly inhibit oral squamous cell carcinoma proliferation through direct cell-contact mechanisms (Ji et al., 2016). These collective properties position GMSCs as promising candidates for developing novel cell-based therapies targeting inflammatory, degenerative, and neoplastic pathologies (Shetty et al., 2025).

### 3.6 Stem cells from Apical Papilla (SCAPs)

During odontogenesis, the dental papilla differentiates into pulp-dentin complexes. These undifferentiated mesenchymal cells regulate tooth morphogenesis and demonstrate odontogenic induction capacity in non-dental epithelium. SCAPs, possessing self-renewal capacity and fibroblastic morphology, are isolatable from developing root apices (Sonoyama et al., 2006). SCAPs exhibit superior proliferative kinetics compared to DPSCs, with constitutive expression of MSCs markers (STRO-1/CD146) and putative SCAPs-specific antigen CD24 (Liang et al., 2022). Zhang et al. established immortalized SCAP lines (iSCAPs) from murine models, demonstrating Wnt3A-mediated upregulation of alkaline phosphatase (ALP) expression and enhanced osteogenic commitment. Moreover, *in vitro* analyses revealed SCAPs’ tripartite regenerative competence: enhanced migratory capacity, matrix mineralization potential, and neo-tissue formation ability, positioning them as prime candidates for craniofacial tissue engineering applications (Bakopoulo et al., 2011). In addition, under hypoxic conditions, SCAPs demonstrate enhanced neurogenic differentiation through upregulation of neuron-specific enolase (NSE), vascular endothelial growth factor-B (VEGF-B), and glial cell line-derived neurotrophic factor (GDNF), accompanied by increased expression of neuronal lineage markers. GDNF overexpression coupled with neuronal marker activation potentiates neurodifferentiation capacity of SCAPs (Vanacker et al., 2014). These neurogenic properties suggest therapeutic potential for SCAPs in neurodegenerative disorder management.

In summary, DPSCs, PDLSCs, and DFSCs have either entered or are approaching the stage of large-scale clinical trials or application. Compared to other dental-derived stem cells, this progress primarily stems from their superior tissue accessibility and well-defined therapeutic indications—particularly in the treatment of periodontitis. With further expansion of application scope and in-depth investigation into their mechanisms of action, other types of dental-derived stem cells also hold promise for future clinical translation.

## 4 Comparison of the properties of dental stem cells with other sources of stem cells

### 4.1 Comparison of sources and access

Mesenchymal cells derived from different germ layers show different characteristics. In addition to DSCs, a wide range of MSCs sources have been extensively explored for regenerative medicine. These provide useful reference points for evaluating the potential of DSCs.

BMSCs are the most established and historically applied stem cell type, serving as a benchmark for osteogenesis and immunomodulation (Isobe et al., 2016). However, bone marrow aspiration is invasive, and cell quality declines with donor age (Brunello et al., 2022; Derubeis and Cancedda, 2004), which may affect their application in clinical translation. Compared with DSCs, BMSCs retain robust osteogenic ability but show weaker neurogenic and angiogenic potential (Arthur et al., 2008).

ADSCs can be easily harvested in large quantities with minimal invasiveness. They secrete abundant trophic and angiogenic factors, supporting soft tissue repair (Hutchings et al., 2020). Nevertheless, the performance of ADSCs in angiogenic gene expression, late osteogenesis, proliferation and migration is not as good as some DSCs, such as DPSCs (D’Alimonte et al., 2017; Jin et al., 2019).

UCMSCs are widely utilized allogeneic stem cells with robust differentiation potential (McElreavey et al., 1991). The abundance of these cells in umbilical cord tissue allows for relatively easy collection without harm to neonates. UCMSCs are considered more primitive than BMSCs and exhibit stem cell properties closer to embryonic stem cells (Yoon et al., 2013; Salehinejad et al., 2012). However, their immunomodulatory capacity declines with *in vivo* aging, which may limit their clinical application (Paladino et al., 2017).

Induced pluripotent stem cells (iPSCs) possess unlimited proliferative and differentiation capacity (Takahashi et al., 2007). Dental-derived iPSCs have been successfully generated from DPSCs (El Ayachi et al., 2018), SHEDs, SCAPs (Yan et al., 2010), oral mucosal fibroblasts (Miyoshi et al., 2010), and gingival fibroblasts (Egusa et al., 2010). Notably, oral tissue-derived cells exhibit higher reprogramming efficiency than conventional human dermal fibroblasts (Yan et al., 2010). However, the reconstruction of periodontal supporting tissues using iPSCs remains challenging. In addition, concerns regarding tumorigenicity, genetic instability, and high production costs continue to hinder clinical translation (Takahashi et al., 2007; Nakagawa et al., 2014).

Overall, BMSCs, ADSCs, UCMSCs, and iPSCs each provide valuable insights and benchmarks for regenerative medicine, supported by varying levels of preclinical and clinical evidence. DSCs combine several practical advantages, including relatively non-invasive harvest and promising neurogenic and immunomodulatory properties. A clearer contrast is shown in Table 1. However, the characteristics and functions of various MSCs may be different due to donor age, site and other factors. Meanwhile, their long-term safety, scalability, and comparative efficacy remain to be established in large, well-designed clinical trials, especially for DSCs, which are almost only in early clinical research. Future research should therefore aim to directly compare DSCs with established stem cell sources across different disease models, to clarify whether DSCs can become broadly adopted in clinical practice.

**TABLE 1:** Comparison of the origin and characteristics of stem cells from different tissues and germ layers.

Comparison dimension	DSCs	BMSCs	ADSCs	UCMSCs	iPSCs
Source Organization	Deciduous exfoliated teeth pulp (SHED), Permanent tooth pulp (DPSCs), Apical papillae (SCAPs), Periodontium (PDLSCs)etc.	Bone marrow (ilium, sternum)	Adipose tissue (obtained by liposuction)	Umbilical cord Warton’s glue or perivascular tissue	Reprogramming of somatic cells (e.g., skin cells) to obtain
Germline Origin	Ectoderm (neural crest-derived mesenchyme)	Mesoderm (cell lineage in embryology)	Mesoderm (cell lineage in embryology)	Mesoderm (cell lineage in embryology)	Dependent on somatic cell source
Acquisition Method	Non-invasive (natural loss of deciduous teeth, orthodontic extractions, periodontal surgical waste)	Invasive (bone marrow aspiration with local anesthesia)	Invasive (liposuction, possible postoperative infection)	Collected at the time of delivery of a newborn (one-time opportunity only)	*In vitro* reprogramming (requires complex laboratory operations)
Age Dependence	All ages (deciduous teeth: 6–12 years; permanent teeth and periodontium: adolescents to adults)	The older the donor, the lower the stem cell activity	Level of obesity affects stem cell content	Accessible only at the neonatal stage	No age limit, but reprogramming efficiency decreases with donor age
Ethical Question	None (autologous sources, medical waste reuse)	Low	Low	Low	Low (but reprogramming may be tumorigenic)
Immuno-genicity	Low (autologous storage avoids rejection; high tolerance for allogeneic use)	Low (but matching required for allografts)	Low (allografts may require immunosuppression)	Low (but long-term storage may affect cell activity)	Autologous source avoids rejection, but reprogrammed cells may be abnormal
Storage and Sustainability	High (deciduous teeth, wisdom teeth can be frozen for years; mature “tooth banking” system)	Requires fresh isolation or short-term freezing	Adipose tissue can be frozen, but stem cell activity decreases with freezing time	Requires freezing at delivery, costly for long-term storage	Cryopreservation technology mature, but reprogrammed cell stability to be verified
References	(Gronthos et al., 2000; Ma et al., 2019; Seo et al., 2004; Wang et al., 2022; Chen et al., 2025a; Guo et al., 2017) (Morsczeck et al., 2023; Miura et al., 2003; Zeng et al., 2020; Zhang et al., 2009; Bustos et al., 2014; Sonoyama et al., 2006; Liang et al., 2022)	(Derubeis and Cancedda, 2004)	(Jin et al., 2019)	(Salehinejad et al., 2012)	(Takahashi et al., 2007; El Ayachi et al., 2018; Yan et al., 2010)

### 4.2 Comparison of proliferation characteristics

Compared with other sources of stem cells, DSCs demonstrate enhanced self-renewal potential and maintain more stable proliferative properties under diverse microenvironmental conditions. First, DSCs exhibit better basal proliferative capacity, with their proliferative activity exceeding that of traditional MSCs in some studies. For example, GMSCs demonstrate greater expansion potential, with significantly higher proliferation rates and population expansion numbers compared to BMSCs (Zhang et al., 2009). Studies have shown that SCAPs possess shortened cell cycles and elevated expression of proliferation-related markers, such as proliferating cell nuclear antigen (PCNA) and proliferation marker protein Ki-67 (Qu et al., 2021). The capacity of SHED to proliferate and migrate is greater than that of human UCMSCs (Yang et al., 2020). Nakamura et al. observed similar results by comparing SHEDs, DPSCs and BMSCs’ proliferation rates and gene expression profiles, it was found that SHEDs exhibited significantly higher proliferation efficiency than the other two (*p* < 0.05). This enhanced proliferation may be associated with upregulated expression of genes related to cell proliferation pathways in SHEDs, including various cytokines such as fibroblast growth factor (FGF) and Transforming Growth Factor-beta (TGF-β) (Nakamura et al., 2009). Comparing DPSCs and BMSCs from the same source, DPSCs had higher proliferation rates, mineralization potential, and clone formation. In addition, they had a higher number of stem/progenitor cells compared to the total cell population (Alge et al., 2010). In contrast to BMSCs that undergo proliferation decay after successive passages, DSCs maintain long-term proliferative stability. For example, both SHEDs and DPSCs retain stable expansion capacity at passage 15 (mean population doubling time [PDT]: 25–30 h), whereas BMSCs lose this capacity by passage 8. Beyond passage 8, BMSCs exhibit prolonged PDT (>40 h), flattened morphology, and significantly reduced Ki-67 expression (Shi et al., 2020).

Overall, DSCs performed better than BMSCs and UCMSCs in long-term passage and high proliferation efficiency, and this stability gives them an advantage in regenerative medicine where long-term cell expansion is required. However, the current study still has the effect of donor differences and heterogeneity of culture conditions, and further standardization is needed.

### 4.3 Comparison of immunomodulatory properties

**FIGURE 2:**
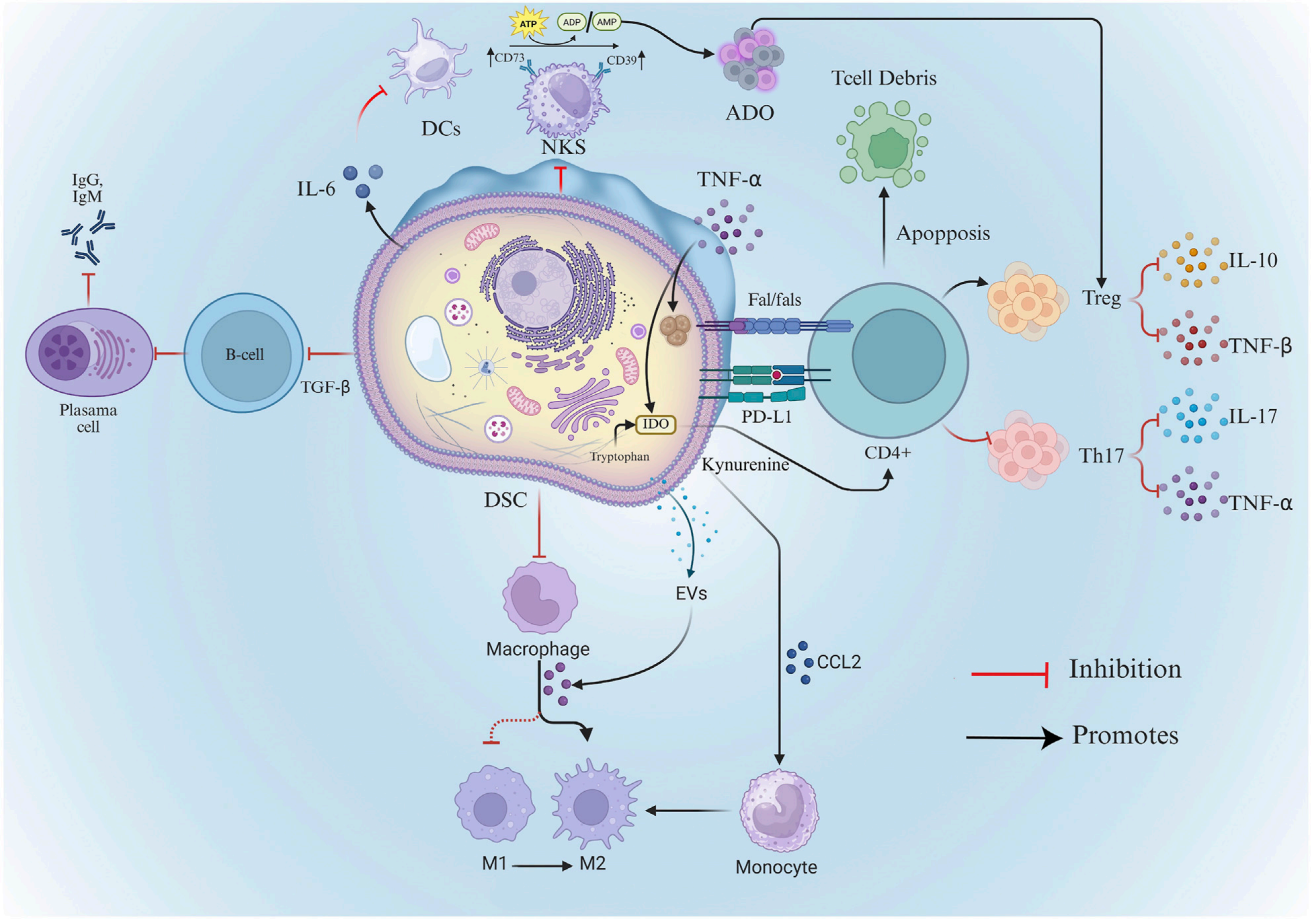
Mechanisms of DSC’s immunomodulatory capacity. DSCs, Dental Stem Cells; Treg, Regulatory T Cells; TGF-β, Transforming Growth Factor-beta; TNF-α, Tumor Necrosis Factor-alpha; DCs, Dendritic Cells; ADO, Adenosin; ATP, Adenosine Triphosphate; ADP, adenosine diphosphate; AMP, Adenosine monophosphate; Evs, extracellular vesicles; NKs, Natural Killer Cell; IDO-Indoleamine-2,3-dioxygenase.

DSCs have low immunogenicity, and existing studies have confirmed that DSCs used in rats did not show immune rejection, and can significantly improve tissue regeneration and repair capacity (Peishan et al., 2023). Moreover, due to their strong immunomodulatory properties, DSCs can inhibit allogeneic transplantation-associated T lymphocytes through direct cell-to-cell contact or through secretion of cytokines or soluble factors such as immunoreceptors or extracellular vesicles (Liu et al., 2015) (Figure 2). This excellent property may be related to the various bacterial flora in the oral cavity and the frequent exposure to inflammatory responses (Wada et al., 2009; Paganelli et al., 2021).

Similar to MSCs from other tissue sources, DSCs secrete immunomodulatory factors including prostaglandin E2 (PGE-2), indoleamine-2,3-dioxygenase (IDO) and TGF-β. These factors regulate innate/adaptive immunity and complement systems, enabling DSCs to suppress excessive inflammation and maintain tissue homeostasis (Lee et al., 2016; Magalhães et al., 2021). For instance, GMSCs upregulate immunosuppressive factors (e.g., IDO, iNOS [inducible nitric oxide synthase], COX-2 [cyclooxygenase-2]) and specifically inhibit peripheral blood lymphocyte proliferation in response to inflammatory cytokines (Zhang et al., 2009; Ding et al., 2010a).

However, in contrast to MSCs from other sources, DSCs exhibit distinct expression patterns of immunomodulatory factors. For instance, SCAPs secrete higher levels of chemotactic and metabolic regulatory proteins than BMSCs (Yu et al., 2016). Similarly, compared with BMSCs, DPSCs can secrete more immunosuppressive factors (e.g., TGF-β1, HGF, IL-10, and IL-13), and have a stronger inhibitory ability against activated T cells (Matsumura-Kawashima et al., 2021). Separately, the conditioned medium from stem cells of human exfoliated deciduous teeth (SHEDs-CM) was found to contain a higher level of the ectodomain of sialic acid-binding Ig-like lectin-9 (ED-Siglec-9) than BMSC-CM. This specific factor demonstrated significant therapeutic efficacy in a mouse model of autoimmune encephalomyelitis by inducing anti-inflammatory M2 macrophages (Shimojima et al., 2016). And compared with BMSCs-CM and ADSCs-CM, SHEDs-CM had higher levels of hepatocyte growth factor (HGF) expression, which plays an important role in SHEDs-CM treatment of myocardial infarction after ischemia-reperfusion, especially in terms of improvement of infarct size and inhibition of cardiomyocyte apoptosis (Yamaguchi et al., 2015).

Secondly, DSCs and BMSCs exhibit divergent abilities in regulating T cell differentiation (Demircan et al., 2011). For example, DSCs can mediate immunoregulatory responses through T cell death. Similar to BMSCs, DPSCs can activate the caspase cascade through the binding of Fas and FasL. This activation leads to T cell apoptosis, which subsequently stimulates macrophages to produce high levels of TGF-β. The increased secretion of TGF-β enhances immune tolerance (Akiyama et al., 2012) and mitigates inflammation-associated tissue damage. Notably, this mechanism has been shown to improve therapeutic efficacy in a mouse model of colitis (Zhao et al., 2012).

DSCs can promote T cell differentiation. DPSCs can induce immune tolerance by increasing the number of local regulatory T cells (Treg) *in vitro* and in a mouse allogeneic skin graft model also (Hong et al., 2019). In addition to promoting Treg differentiation, DPSCs also influence the polarization of CD4^+^ T cells through paracrine mechanisms. Exosomes—small extracellular vesicles (EVs) secreted by stem cells—have emerged as key mediators of this process. These nanosized vesicles carry bioactive molecules such as proteins, lipids, and RNAs, enabling targeted immunoregulatory effects. Notably, exosomes derived from DPSCs (DPSCs-Exo) exhibit stronger immunomodulatory capacity compared to those from BMSCs. They suppress the differentiation of CD4^+^ T cells into pro-inflammatory Th17 cells, reduce the secretion of IL-17 and TNF-α, and simultaneously promote the polarization of CD4^+^ T cells toward the Treg phenotype. This exosome-mediated pathway contributes significantly to establishing immune tolerance and controlling inflammation (Lujun et al., 2019).

Finally, DSCs demonstrate notable distinctions in their immunomodulatory mechanisms compared to MSCs from other sources. DSCs expressed more CD39 than BMSCs and were able to produce more adenosine (ADO) from adenosine triphosphate (ATP), which inhibited the proliferation of CD3^+^ T cells and promoted the generation of CD4^+^CD25+FoxP3+CD39^+^CD73^+^ Tregs. This study demonstrated that DSCs were able to utilize the adenopurinergic pathway as an immunomodulatory mechanism, which was more effective than BMSCs (Poblano-Pérez et al., 2023). PGE-2 was found to play an important role in PDLSC-mediated immunomodulation and periodontal tissue regeneration in a small porcine model of periodontitis (Ding et al., 2010b). Similar to them, GMSCs can significantly inhibit contact hypersensitivity (CHS) by suppressing mast cell pro-inflammatory cytokine expression at source through PGE2 (Su et al., 2011). TGF-β eliminates IgM and IgG production through allogeneic activation of responder B lymphocytes. TGF-β secreted by DPSCs has been shown to be a major factor in inducing immunosuppression in acute allogeneic immune responses (Kwack et al., 2017) and can increase CD4^+^CD25+Foxp3+ regulatory T cells (Hong et al., 2019) and inhibit the proliferation of allogeneic lymphocytes (Hossein-Khannazer et al., 2019). Furthermore, PDLSCs suppress B cell activation via PD-1/PD-L1 ligand-receptor interactions during cell-cell contact. When co-cultured with normal B cells, it can inhibit their proliferation, differentiation, and migration, and reduce the secretion of IgM and IgG (Liu et al., 2013). GMSCs additionally reduce dendritic cell (DC) and mast cell (MC) infiltration, consequently suppressing production of multiple inflammatory cytokine (Su et al., 2011).

In contrast, the immunomodulatory mechanisms of BMSCs and ADSCs are more dependent on IDO and PGE-2, while DSCs also show special adaptability derived from the oral microenvironment (Table 2). This suggests that MSCs from different sources may have differential applicability in specific disease models.

**TABLE 2:** Comparative immunomodulatory factor secretion profiles of MSC sources.

MSC	IL-10	TGF-β	Ido	PGE2	References
**DSCs (DPSCs, PDLSCs, GMSCs)**	↑↑	↑	↑	↑	Mohan Kumar and Thakkar (2024)
**BMSCs**	↑	↑↑	↑↑	↑↑	Lin et al. (2023a)
**ADSCs**	↑	↑	↑	↑↑	Sandra Sari et al. (2023)
**UCMSCs**	↑↑	↑↑	↑	↑	Hussien et al. (2025)

↑↑ = high level/evidence; ↑ = moderate level/evidence. Levels reflect relative strength of evidence based on multiple peer-reviewed studies rather than absolute quantitative values.

However, these experiments based on simplified or static conditions may not fully replicate the complexity of the *in vivo* immune microenvironment. Moreover, the heterogeneity among different types of DSCs suggests that their immunomodulatory capabilities may be inconsistent, and the molecular basis for these differences remains unclear. In addition, although paracrine signaling through EVs is considered an important mechanism of immune regulation, few studies have systematically compared EVs composition or activity of DSCs from the same individual or from different sources. Therefore, future studies should focus on standardising *in vitro* experimental conditions and elucidating the signaling pathways responsible for DSCs-mediated immune regulation to more accurately characterize the immune properties of DSCs.

### 4.4 Comparison of osteogenic capacity

Multiple studies demonstrate the osteoregenerative capacity of DSCs (Omori et al., 2015; Ge et al., 2012a). This is dependent on its promotion of osteogenesis, inhibition of osteoclastogenesis, and regulation of inflammation during bone healing.

Firstly, the excellent osteogenic ability of DSCs is due to the high expression of osteogenic genes. ALP is well-known critically involved in osteogenesis. Alge et al. compared matched donor-derived DPSCs and BMSCs, revealing significantly higher ALP activity in DPSCs (Alge et al., 2010). SHEDs-CM induces BMSCs and osteoblast precursor cells (MC3T3-E1 cells) osteogenic differentiation with higher levels of mRNA expression of osteogenic differentiation markers (ALP, OCN, Runx2) (Kunimatsu et al., 2022). Furthermore, DSCs can secrete higher levels of VEGF (Kushiro et al., 2021). VEGF modulates osteogenic signaling, stimulates osteoblast chemotaxis/differentiation, and promotes angiogenesis in bone defects, thereby facilitating osteogenesis (Tarkka et al., 2003). SCAPs maintain proliferative stability under hypoxia while upregulating osteogenic/angiogenic genes. Notably, SCAPs enhance sustained VEGF-A production (Vanacker et al., 2014). Granulocyte colony-stimulating factor (G-CSF)-mobilized DPSCs express higher VEGF-A than BMSCs and ADSCs (Murakami et al., 2015).

Secondly, DSCs help to reduce bone mass loss by inhibiting osteoclast activity. OPG is a well-established inhibitor of osteoclast differentiation. Kanji et al. demonstrated that DPSCs suppress osteoclastogenesis through OPG secretion and AKT signaling inactivation in myeloid cells (Kanji et al., 2021). SHEDs-CM contains 2.8-fold higher OPG levels than BMSCs-CM. Additionally, ED-Siglec-9—a characteristic component—more potently inhibits osteoclastogenesis *in vitro*. Combined with anti-inflammatory properties, ED-Siglec-9 demonstrates significant therapeutic efficacy in rheumatoid arthritis (RA) mouse models (Ishikawa et al., 2016). Furthermore, Lu et al. (Lu et al., 2023) revealed that PDLSCs-derived exosomes suppress osteoclastogenesis via the miR-31–5p/eNOS signaling pathway *in vitro*. According to previous studies, GMSCs-derived EVs significantly attenuate inflammatory bone loss and reduce TRAP-positive osteoclasts in periodontitis models. This effect is mediated by exosomal miR-1260b, which inhibits RANKL signaling via Wnt5a suppression, thereby blocking osteoclastogenesis (Nakao et al., 2021).

Finally, relying on the powerful immunomodulatory ability of DSCs mentioned earlier, it helps them to better repair bone defects. In addition to the immunological properties already mentioned earlier, in terms of osteogenesis, DPSCs-CM contains elevated levels of anti-inflammatory cytokines—including IL-10, IL-13, and TGF-β1—compared to BMSCs-CM (Ogata et al., 2021; Bousnaki et al., 2022). Moreover, SHEDs rescue dysfunctional BMSCs by suppressing IL-17 in recipient bone marrow, restoring osteoblast-osteoclast equilibrium. In addition, Ge et al. demonstrated that GMSCs maintain stable proliferation and trilineage differentiation capacity under inflammatory conditions (Ge et al., 2012b). Similarly, TNF-α exposure minimally inhibited the osteogenic potential of GMSCs compared to other DSCs (Tian et al., 2022). But meanwhile, DSC properties exhibit microenvironment-dependent heterogeneity (Zhou et al., 2020). Comparison of the properties of PDLSCs from healthy and “inflamed” patients revealed that the latter showed greater migratory potential and higher proliferative capacity, but a reduced capacity for osteogenic differentiation (Tang et al., 2016). It has been suggested that this may be related to the upregulation of Ubiquitin C-terminal hydrolase L1 (UCHL1) in inflamed periodontal tissues, which activates the negative feedback regulation of the BMP2/Smad signaling pathway, consequently impairs the osteogenic capacity of PDLSCs, and accelerates bone resorption in alveolar bone (Lin L. et al., 2023).

DSCs, particularly DPSCs and PDLSCs, exhibit robust osteogenic differentiation potential, generally comparable to BMSCs (Zhao et al., 2023). ADSCs also display osteogenic ability but often generate less mineralized tissue (Truchan et al., 2025), while UCMSCs contribute to bone repair primarily via paracrine effects rather than direct osteogenesis (Yang X. et al., 2024). Overall, BMSCs remain the benchmark for osteogenic studies, yet DSCs offer unique tissue-specific compatibility in craniofacial contexts, which may facilitate improved integration and regeneration. In several studies, DSCs exhibit comparable or even superior mineralization capacity to BMSCs, yet these observations are not always consistent across research groups, suggesting potential methodological bias or variation in osteoinductive stimuli. Moreover, the intrinsic mechanisms governing osteogenic differentiation in DSCs remain incompletely understood. For example, signaling pathways such as BMP/Smad, Wnt/β-catenin, and MAPK have been implicated, but the relative contribution of each pathway may differ among DSCs types. Additionally, most *in vitro* assays rely on short-term mineral deposition as the endpoint, which may not accurately reflect long-term matrix maturation and biomechanical stability. To strengthen the evidence base, future studies should actively explore the relevant osteogenic regulators that determine the lineage-specific differentiation of DSCs.

### 4.5 Comparison of neurological capacity

DSCs derive from the neural crest—an ectodermal progenitor population that generates peripheral nervous system cells (Martens et al., 2013). Because of its homology with nerve cells, DSCs exhibit neural characteristics including neural marker expression, neurotrophic factor secretion, and differentiation into functional neurons (Arthur et al., 2009; Király et al., 2011). Naive DPSCs express neural markers including nestin, neurofilaments, vimentin, S100, and βIII-tubulin (Karaöz et al., 2010; Kerkis et al., 2006). Increased expression of class III b microtubulin (TUBB3) and microtubule-associated protein 2 (MAP2) in RT-PCR compared to BMSCs confirms that DPSCs and SHEDs exhibit neurogenic potential (Isobe et al., 2016). Meanwhile, the synergistic immunomodulatory and anti-inflammatory properties of DSCs (Cooper et al., 2019) enhance their suitability for neural tissue engineering over other MSCs.

DSCs promote neuronal growth, survival, and protect neuronal cells through higher levels of neurotrophic factors, primarily comprising brain-derived neurotrophic factor (BDNF), nerve growth factor (NGF), and neurotrophin-3 (NT-3) (Qiu et al., 2025). G-CSF-mobilized DPSCs exhibit elevated neurotrophic factor expression and enhanced trophic effects on cell migration/apoptosis *versus* BMSCs or ADSCs (Murakami et al., 2015). Apical complex-derived DPSCs express higher NT-3 and NT-4 in conditioned media than dental apical complex cells (DACC) during odontogenic differentiation (Joo et al., 2018). In particular, DPSCs-CM was able to induce greater neurite growth in neuroblastoma. Comparative analysis revealed DPSCs secrete 3- to 10-fold higher neurotrophic factors (e.g., NGF, BDNF) than BMSCs or ADSCs in co-culture systems, as quantified by RT-qPCR, microarray, and ELISA (Mead et al., 2014). In other study, SHEDs exhibit better neural process outgrowth promotion compared to UCMSCs (Yang et al., 2020). Sakai et al. demonstrated therapeutic efficacy of DPSCs and SHEDs transplantation in rat spinal cord injury models (Sakai et al., 2012). Additionally, Tomokiyo et al. revealed that hPDLSC-derived neural crest cells (NCCs) exhibit greater pluripotency than NCCs differentiated from foreskin fibroblast-iPSCs (Tomokiyo et al., 2017).

To sum up, SHEDs and DPSCs demonstrate better neurogenic potential compared with other MSC sources, reflecting their neural crest origin. They express neuronal markers, promote neurite outgrowth, and support angiogenic processes, which collectively favor neurovascular regeneration. In contrast, BMSCs and ADSCs show more limited neurogenic differentiation (Isobe et al., 2016), and UCMSCs mainly exert neuroprotective roles through trophic and immunomodulatory factors (Yang et al., 2020). Thus, DSCs provide a distinct advantage for neuroregenerative applications within dental and craniofacial tissues. However, as most studies on the neurogenic potential of DSCs rely on various animal models, significant gaps remain in our understanding of the underlying mechanisms. For instance, although DPSCs and SHEDs have been shown to differentiate into neural-like cells *in vitro*, their ability to integrate into existing neural networks *in vivo* is still uncertain, highlighting the need for further research.

**FIGURE 3:**
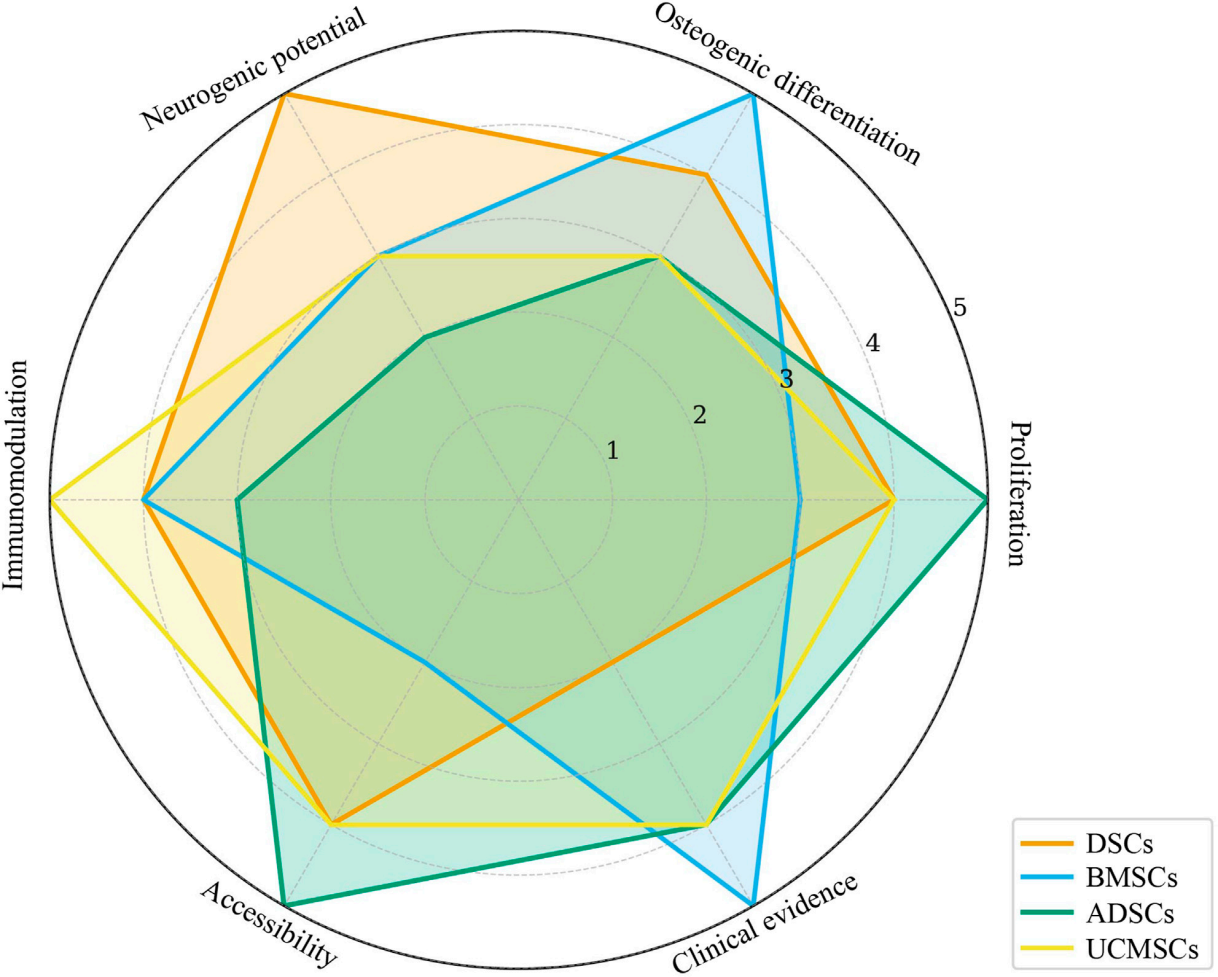
Radar chart summarizing key biological properties of MSCs from different sources. DSCs, Dental Stem Cells; BMSCs, Bone Marrow Stem Cells; ADSCs, Adipose-derived Stem Cells; UCMSCs, Umbilical Cord Mesenchymal Stem Cells.

Figure 3 shows the radar map of the characteristics of MSCS from each source.

## 5 Advantages of dental stem cells in the treatment of related diseases

**FIGURE 4:**
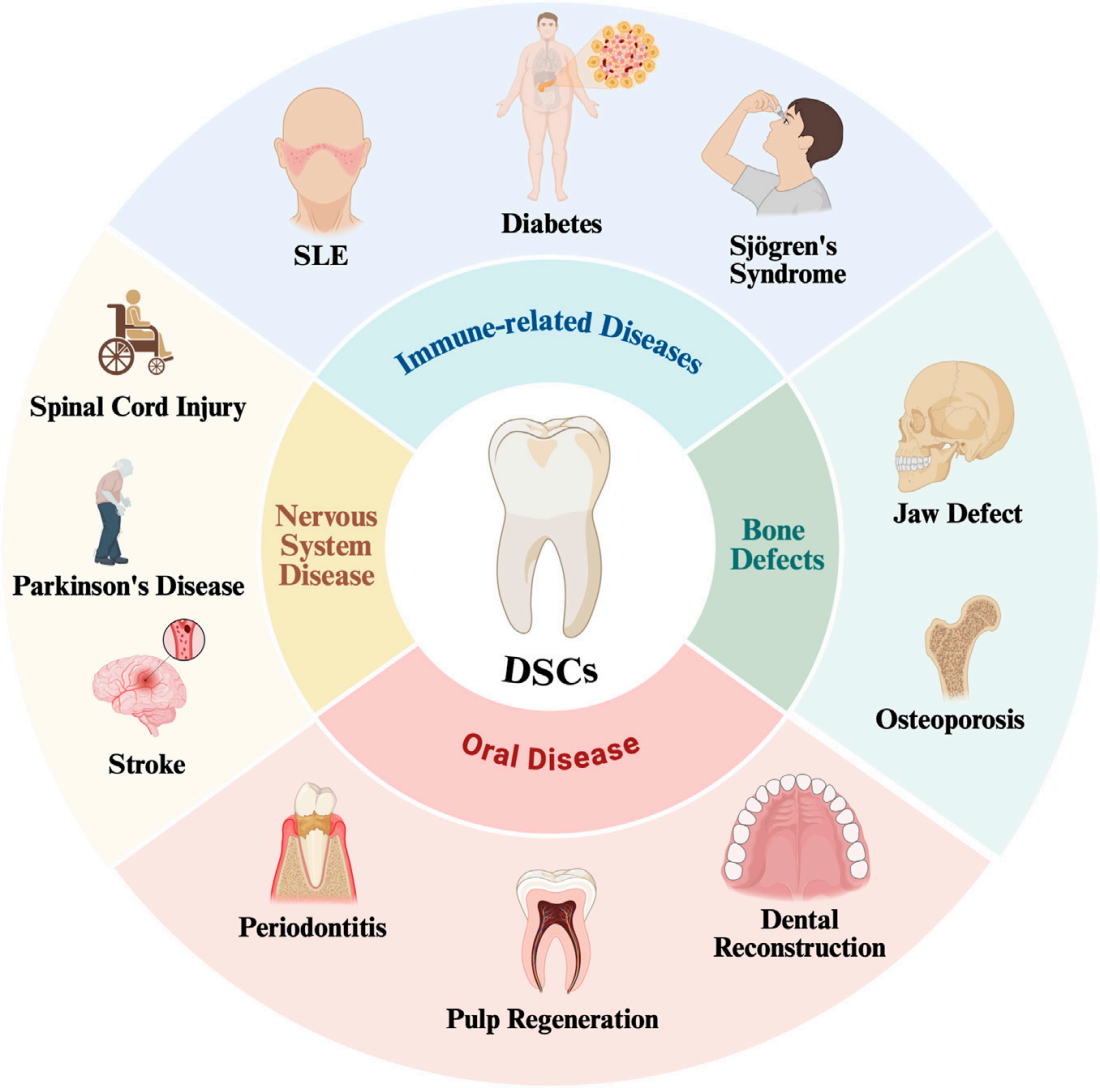
DSCs have been used in the treatment of a variety of diseases SLE, Systemic Lupus Erythematosus.

Due to their excellent properties, DSCs can be used to treat a variety of diseases, including immune-related diseases, osteogenesis diseases, nervous system diseases and oral diseases (Figure 4).

### 5.1 Immunomodulation and immune-related diseases

Most immune disorders result from dysregulated immune responses causing imbalance and chronic inflammation. These include Sjögren’s syndrome, SLE, inflammatory bowel disease (IBD), and diabetes mellitus (Shoda et al., 2023). Current treatments for these diseases rely on the use of non-antigen specific, broad-spectrum immunosuppressive or immunomodulatory compounds, but these drugs can also impair the normal immune response and even increase the risk of infection and malignancy (Montaño et al., 2021). DSCs exhibit low immunogenicity and potent immunomodulatory capacity, enabling therapy for diverse immune-mediated diseases. DPSCs-CM ameliorates Sjögren’s syndrome by suppressing Th17 differentiation and enhancing Treg generation in splenic tissue. Compared to BMSCs, DPSCs demonstrate better T cell inhibition and anti-apoptotic effects, improving salivary secretion in Sjögren’s syndrome (Matsumura-Kawashima et al., 2021). The pioneering clinical study demonstrated that stem cells from SHEDs transplantation improves glucose metabolism and islet function in type 2 diabetes patients (Li W. et al., 2021). On the one hand, stem cells can be induced to differentiate into insulin-like cells, and on the other hand, stem cells can be used to modulate the immune response against pancreatic antigen tolerance (Xu et al., 2020). GMSCs inhibit allergic responses and enhance tissue regeneration/wound healing, offering novel therapies for autoimmune diseases including RA and IBD (Tian et al., 2022; Lu et al., 2019).

In addition, BMSCs from SLE patients exhibit impaired proliferation, increased senescence/apoptosis, and reduced immunosuppressive capacity (Sun et al., 2007). Conversely, Yamaza et al. demonstrated successful application of SHEDs in SLE mouse models. SHEDs reduced peripheral Th17 cells, restored bone trabecular structure, and inhibited osteoclast activity, collectively ameliorating SLE pathology (Yamaza et al., 2010). Therefore, SHEDs may serve as new seed cells for the treatment of SLE patients. Meanwhile, DPSCs demonstrate enhanced efficacy against severe COVID-19 pneumonia compared to other stem cell sources (Zayed and Iohara, 2020).

Although DSCs exhibit strong immunomodulatory properties, and *in vitro* studies have shown promising results, the reported therapeutic effects vary. Additionally, most studies have been conducted on small animal models, which may limit the generalizability of these findings to human clinical settings. For example, some studies focus on inflammatory conditions in rodents, but the results may not fully reflect the complexity of the human immune system. Future research should aim to use animal models that more closely resemble human lineage, larger sample sizes, and attempt to include multicenter clinical trials to better assess the immunomodulatory potential of DSCs.Although *in vitro* studies have shown promising results, most evidence remains at the animal level. Most of the existing clinical trials are phase I/II, and the sample size is limited, which is not enough to support widespread use.

### 5.2 Treatment of bone defects

Alveolar and cranial bone defects typically arise from congenital malformations, trauma, or tumor resection (Lin et al., 2024). Current treatments mainly include the use of autologous bone grafts (Mahardawi et al., 2023) and allogeneic bone and synthetic material grafts, but their usual limitations such as insufficient autologous bone volume, allogeneic rejection, or suboptimal osteoinductive capacity (Ashfaq et al., 2024). Stem cell advancements offer novel strategies for bone regeneration therapies.

As mentioned previously, DSCs have good osteogenic properties. Compared with ADSCs, UCMSCs, and amniotic membrane mesenchymal stem cells (AMSCs), DPSCs have the best therapeutic effect on postmenopausal osteoporosis, and can preserving bone mass, This advantage may stem from DPSCs’ unique capacity to modulate Th17/Treg balance and macrophage M1/M2 polarization (Li et al., 2024). In addition, Yu et al. compared the treatment of critical-size cranial defects in immunodeficient rats with BMSCs and PDLSCs and found that the PDLSCs had a stronger bone regeneration ability (Yu et al., 2014). Besides, in a study in 2009, DPSCs isolated from the patient’s maxillary third molar then co-cultured with collagen scaffolds, and a significant increase in the amount of bone repair in the extraction sockets was observed at 3 months postoperatively compared with that in the control group (d’Aquino et al., 2009). Three-year follow-up revealed denser, more homogeneous vascularized bone regeneration in DPSC-collagen scaffold treated sites, confirming the long-term efficacy of DPSCs-mediated osteoinduction (Giuliani et al., 2013).

In preclinical and clinical studies, BMSCs have consistently shown efficacy in bone defect repair and remain widely applied in alveolar cleft and craniofacial bone augmentation (Bajestan et al., 2017; Luo et al., 2022). DSCs have demonstrated comparable outcomes in periodontal and alveolar regeneration, with additional benefits in microenvironmental matching. ADSCs have been explored for periodontal bone repair but generally yield fewer stable outcomes than BMSCs or DSCs (Truchan et al., 2025). UCMSCs-based therapies show promise through immunomodulation and angiogenesis but require further validation. Together, these findings suggest DSCs hold a context-specific advantage for oral and maxillofacial bone regeneration, complementing the broader clinical experience of BMSCs.

### 5.3 Diseases of the nervous system

In recent years, various types of neurological diseases are highly prevalent, such as: Alzheimer’s disease, Parkinson’s disease, stroke, spinal cord injury and peripheral nerve injury. Advances in stem cell therapy have increased the potential for treating neurological diseases with stem cells (Ibarretxe et al., 2012). Although neural stem cells (NSCs) can be differentiated into neurons, astrocytes and oligodendrocytes and help to delay neuronal or other neural cell damage by using self-renewal and neural differentiation (Stenu et al., 2015), their procurement from human brain tissue remains challenging and invasive. Consequently, DSCs—owing to their homology with NSCs—have emerged as promising candidates for neurodegenerative disease research.

Several animal studies demonstrate the reparative potential of DSCs in peripheral and central nerve injuries. For example, DPSCs, SHEDs, and SCAPs show therapeutic efficacy in spinal cord injury (Zhou et al., 2023), Alzheimer’s disease (Howlader et al., 2024), Parkinson’s disease (Chen et al., 2024), stroke (Miao et al., 2024), etc. Sharma et al. report that DPSCs may have a better therapeutic potential for Parkinson’s disease than BMSCs due to their better propensity for neural differentiation and neuronal regeneration capacity (Sharma et al., 2022). And because it may be able to transform into dopaminergic neuronal cells under hypoxic conditions *in vitro*, it showed better behavioral improvement in a rat model of Parkinson’s disease (Greene, 2009).

Compared with BMSCs, intravenous transplantation of DPSCs resulted in a similar state of functional repair but reduced infarct volumes observed in a rat stroke model (Song et al., 2017). DPSCs overexpressing stem cell growth factor can modulate the inflammatory response and blood-brain barrier permeability during the acute phase of stroke, increasing their neuroprotective effects and preventing brain injury after ischemia/reperfusion (I/R) (Sowa et al., 2018). Wu et al. compared the therapeutic effects of DPSCs and PDLSCs on stroke by using PKH26 staining to tracer the migration of stem cells, and found that the latter showed a more pronounced PKH26 fluorescence labeling signal and more significant efficacy in promoting neurological recovery (Wu et al., 2020). DPSCs-Exo attenuated cerebral edema, cerebral infarction and neurological impairment in cerebral I/R mice, and it significantly inhibited I/R-mediated expression of TLR4, MyD88 and NF-κB expression and increased anti-inflammatory levels by inhibiting the HMGB1/TLR4/MyD88/NF-κB pathway (Li S. et al., 2021). In addition, because of the higher expression level of neurotrophic factors in DPSCs, their co-culture with retinal ganglion cells (RGCs) significantly promoted RGCs survival and neurite regeneration, and the transplantation of DPSCs induced a more pronounced promotion of axon regeneration and neuroprotective effects compared with BMSCs (Mead et al., 2017).

DSCs-based therapies, particularly those employing SHEDs and DPSCs, have shown favorable results in models of nerve regeneration, demonstrating restoration of vascularized and innervated tissues. BMSCs and ADSCs have also been tested in neurodegenerative disease models but with limited differentiation capacity, relying more on trophic support. UCMSCs exhibit indirect benefits through secretion of neuroprotective and anti-inflammatory mediators. While current clinical evidence remains preliminary, DSCs emerge as an attractive candidate for future neuroregenerative therapies in dental and broader neurological contexts. But the neurorepair efficacy observed in animal models may not fully reflect outcomes in humans. The complexity of the nervous system, along with variations in experimental conditions, such as the use of different neurotrophic factors and scaffold materials, further complicates the translation of these results. Therefore, more reliable studies, particularly clinical trials involving human patients, are needed to evaluate the true neurogenic potential of DSCs in humans.

## 6 Unique benefits of oral disease treatment

Owing to their inherent homology and microenvironmental niche compatibility with oral tissues, DSCs have been the most extensively studied for the treatment of oral diseases. This section will focus on their specific potential and clinical progress in oral tissue regeneration. There have been many animal experiments using MSCs to treat oral diseases, and representative ones in recent years are shown in Table 3.

**TABLE 3:** Summary of recent animal studies using MSCs in oral and maxillofacial disease models (2023–2025).

Cell type	Material/scaffold	Disease model	Animal	Key outcomes	References
PDLSCs	Antimicrobial peptide-functionalized PDLSCs	Fenestration periodontal defect	Sprague-Dawley rats	Promoted periodontal regeneration and improved oral microbiota	You et al. (2024)
DPSCs	Extracellular vesicles (local injection)	Alveolar bone defect	Sprague-Dawley rats	Upregulated osteogenic markers and enhanced bone repair	He et al. (2025)
SHEDs	Conditioned medium	Condylar chondrocyte inflammation model	Mouse	Altered expression of inflammation-associated molecules in condylar chondrocytes	Xia et al. (2023)
BMSCs	BMSCs + calcium phosphate cement (CPC) composite	Alveolar ridge preservation/tooth extraction socket wound healing	Beagle dogs	Significantly improved alveolar ridge preservation and enhanced bone healing post extraction	Yang et al. (2025)
ADSCs	ADSCs + Demineralized dentin matrix (DDM)	Periodontitis-induced bone defect	Rats	Improved alveolar bone and periodontal ligament regeneration	Sandra Sari et al. (2023)
UCMSCs	Exosome-loaded hydrogel	Ligature-induced periodontitis	Mouse	Reduced alveolar bone loss and promoted a reparative microenvironment	Li et al. (2025)

*Asterisk denotes secretome-based acellular strategies without live cell transplantation.

### 6.1 Periodontitis

Periodontitis is a disease of the oral cavity that has become prevalent in recent years. When treating periodontitis, in addition to the mentioned previously regeneration of alveolar bone, it is necessary to focus on the specific microenvironmental factors within the patient’s mouth. When inflammation is caused by pathologic factors, the pH value in the oral cavity may decrease, leading to a decrease in the osteogenic capacity of BMSCs and DPSCs (Massa et al., 2017). And the regenerative capacity of stem cells will be reduced in the case of localized inflammation. Some researchers tried to insert recombinant human IGFBP5 protein (rhIGFBP5) into PDLSCs to enhance their regenerative ability, which can improve higher osteogenic differentiation ability in an animal model of inbred male minipigs (Han et al., 2017). Meanwhile, the osteogenic capacity of stem cells can be enhanced by co-culturing different stem cells, such as co-culturing BMSCs with PDLSCs to mimic 3D bone tissues *in vitro*, which has stronger performance than a single species of stem cells (Zhang et al., 2016). BMSCs derived from the maxilla can also enhance the osteogenic capacity of PDLSCs *in vitro* (Jin et al., 2016).

In addition, the local inflammatory state can also be regulated by appropriate scaffold design, for example, using calcium alginate hydrogel, into which PDLSCs and BMSCs were incorporated, and both types of stem cells showed good osteogenic differentiation and reduced the degree of inflammation of local tissues in New Zealand rabbits *in vivo* (Chen et al., 2017). Chen et al. conducted the first study of DSCs in clinical trials, a randomized trial using autologous PDLSCs to treat periodontal intraosseous defects, which validated the safety of PDLSCs in clinical applications (Chen et al., 2016). In addition, the phase III clinical trials of DPSCs injection (Liu et al., 2025) and the recently approved human clinical trials of DFSCs injection in the targeted treatment of periodontitis further demonstrated the promising efficacy of DSCs in the treatment of periodontitis.

### 6.2 Gingival recession

Gingival recession is a common oral disease with a high prevalence. It may be caused by several factors, including aggressive brushing, smoking, concentrated occlusal stress, and orthodontic treatment in older adults. GMSCs exhibit therapeutic advantages for gingival recession owing to tissue homology. GMSCs produce and secrete higher amounts of interleukin-1 receptor antagonist (IL-1RA)–expressing small EVs than skin MSCs. These sEVs accelerate the healing of gingival wounds (Kou et al., 2018). Sanchez et al. combined the treatment of localized gingival recession with the addition of stem cells by inoculation of autologous GMSCs into an allogenic collagen matrix with a coronal late-stage flap inoculated, and found that wound healing was better with the addition of stem cells, and that inflammation and postoperative complications associated with gingival surgery were greatly reduced (Sanchez et al., 2022).

### 6.3 Pulp regeneration

When a tooth has irreversible pulpal inflammation due to dental caries or when the pulp is exposed due to trauma, pulpotomy or endodontic treatment is required, but these treatments result in inactivation of the pulp that supplies nutrients to the tooth, which tends to lead to darkening of the color of the tooth in later stages as well as to increased brittleness of the tooth and the risk of fracture (Xie et al., 2021). Therefore, there is an urgent need to find a way to help reestablish active and functional pulp regeneration.

SHEDs and DPSCs have strong angiogenic capacity and neurogenicity, and are for pulp regeneration excellent seed cells due to their homology with pulp tissue (Yang N. et al., 2024; Chen et al., 2021). Xuan et al. implanted ex vivo-expanded autologous SHEDs into pulp necrosis patients, observing regeneration of three-dimensional pulp structures containing odontoblasts, vasculature, and neural networks (Xuan et al., 2018). However, the survival time of DSCs in the root canal is limited. In recent years, advances in biomaterials enable stem cell delivery via scaffold systems for enhanced pulp regeneration. For instance, DPSCs seeded in thermoresponsive hydrogels and implanted into human root canals subcutaneously transplanted in immunodeficient mice formed vascularized pulp-like tissues within 6 weeks. Odontogenic differentiation occurred at dentin-contact sites, with mineralized centers containing CD31^+^ endothelial cells (Itoh et al., 2018). Prescott et al. implanted DPSCs with gelatin scaffolds containing dentin matrix protein-1 (DMP-1) into dentinal cavities of veneers in nude mice, demonstrating pulp-like tissue formation in cavities after 6 weeks (Prescott et al., 2008). Lu et al. cultured SHEDs in odontogenic medium (OM) within photocrosslinkable gelatin methacryloyl (GelMA) hydrogels, observing odontogenesis in subcutaneously grafted root sections. After 8 weeks, mineralized tissue showed elevated dentin sialophosphoprotein (DSPP) and DMP-1 levels *versus* controls (Lu et al., 2024).

A clinical trial study on the feasibility of using DPSCs to replace infected pulp tissue has been conducted in Japan in 2014, which utilized autologous DPSCs to treat irreducible pulpitis in autogenous teeth, and patients had no adverse effects and there was restoration of pulp vitality after 25 weeks (Nakashima and Iohara, 2014). There is also a study in the United States using autologous SHEDs to treat pulp and apical tissues in patients with pulp necrosis in young permanent teeth, focusing on evaluating its efficacy and safety.

### 6.4 Dental reconstruction

Tooth regeneration presents a multifaceted challenge, primarily requiring coordinated regeneration of the alveolar bone-periodontium complex and functional restoration of the pulp-dentin unit. Some studies have seeded on PDLSCs and DFSCs on material scaffolds and applied them to tooth regeneration models in animals, and found that root-periodontal-like tissues were formed (Lu et al., 2024; Guo et al., 2012). Similarly, iPSCs and PDLSCs transplanted into periodontal defect models regenerate periodontal ligament, odontoblasts, and alveolar bone (Chen et al., 2025b). This approach effectively repairs periodontal tissue defects (Seo et al., 2004). And the regenerative aspects of the first two have been described previously.

In addition to this, the utilization of DSCs to regenerate the non-regenerable enamel after abrasion is of great research significance. Kim et al. developed a method in which direct co-culture of hESCs/hiPSCs with Hertwig’s epithelial root sheath/epithelial rests of Malassez successfully generated dental epithelial-like stem cells. Additionally, the iPSC line co-cultured with DPSCs showed increased expression of amelogenic and odontogenic genes. (Kim et al., 2020). CD24a+ SCAP can regenerate functional dentin and neurovascular-like structures in the tooth roots (Chen et al., 2020). In addition to this, iPSCs can be used for the reconstruction of enamel that is theoretically non-regenerative after abrasion, and the ability to differentiate iPSCs into enamel-forming cells was first reported by Arakaki et al. (Arakaki et al., 2012). Subsequent studies show that iPSCs cultured in ameloblast serum-free conditioned medium with BMP-4 differentiate into ameloblast- and odontoblast-like cells (Liu et al., 2016). Thus, iPSCs offer a viable pathway for enamel regeneration.

In general, different sources of stem cells have their own advantages in the treatment of diseases. DSCs have shown unique potential in neurological, immune and oral diseases, while BMSCs and ADSCs have accumulated more clinical experience in bone defect repair. Future studies need cross-source systematic comparison and long-term follow-up to promote the precise and individualized application of stem cell therapy.

## 7 Conclusion and prospects

DSCs have many advantages in addition to the various types of excellent properties mentioned previously. DSCs can be stored for long periods of time. With the invention of storage solution for isolated teeth, treated isolated teeth can be stored at 4 °C for 120 h and can be isolated and cultured to produce active and proliferative DPSCs (Perry et al., 2008). And with the development of cryoprotective solution, to a certain extent, it provides a basis for the long-distance transportation of isolated teeth. Moreover, with the establishment of “tooth banks” in developed countries such as Japan, the United States and Norway, cell sources can be provided for basic research and clinical application of tooth-derived stem cells. More surprisingly, Arora et al. (Arora et al., 2009) showed that the storage cost of SHEDs bank is about 1/3 of that of umbilical cord blood cell bank, which is an important advantage of tooth-derived stem cell bank over other sources of stem cell bank. Though, comparative evidence indicates that while DSCs display strong neurogenic, angiogenic, and immunomodulatory capacities, other MSC sources retain established advantages, particularly in osteogenesis and systemic immune disorders. Accordingly, no single MSC source can be regarded as universally optimal, and their use should be tailored to specific clinical contexts.

Despite encouraging findings, the translation of MSC-based therapies remains constrained by donor heterogeneity, lack of standardized isolation and expansion protocols, and limited long-term safety and efficacy data. As several clinical trials have been registered on www.clinicaltrials.gov. (Table 4), but most clinical studies remain at early phases (I/II) with small cohorts and non-uniform outcome measures. And simultaneously, regulatory divergence across countries further complicates clinical adoption. In the long run, we still need some reliable industry norms to ensure the institutional standardization and standardization of the process of obtaining, producing, and using DSCs (Miguita et al., 2017).

**TABLE 4:** Clinical trials involving DSCs.

Disease	NCT	Stem cell source	Phase/design	Sample size	Status	Primary outcomes	Results/notes
Knee Osteoarthritis	NCT04130100	Allogeneic DPSCs	Phase I/II, randomized, 3-arm (low/high dose vs. hyaluronic acid)	60 (planned)	Recruiting	Pain (VAS), function (WOMAC)	No results posted; exploratory evidence only
COVID-19 (Severe Pneumonia)	NCT04336254	Allogeneic DPSCs	Phase I/II, single-centre, IV infusion	∼20 (planned)	Unknown/not updated	Safety, pulmonary function, survival	No results posted; uncertain completion
Type 1 Diabetes Mellitus	NCT03912480	Allogeneic SHEDs	Early Phase I, open-label	24 (planned)	Recruiting (2024 update)	C-peptide, insulin requirement	No results posted
Type 2 Diabetes Mellitus	NCT03658655	Allogeneic SHEDs	Phase I/II, single-centre	—	Recruiting	Glycemic control, safety	No results posted
Periodontal Intrabony Defects	NCT03638154	DPSCs	Randomized, DPSC vs. PRP	20	Completed	CAL gain, bone fill (6 months)	No registry results; small pilot
	NCT01082822	Autologous PDLSCs	Early phase, controlled	20	Completed	Bone regeneration after third molar extraction	No registry results; early pilot
Periodontitis	NCT01357785	Autologous PDLSCs	Randomized, placebo-controlled	—	Completed/unknown	CAL, probing depth, bone fill	No posted results; early RCT
Periodontal Regeneration	NCT03137979	GMSCs	Interventional, with bone graft	—	Unknown	Periodontal regeneration indices	No registry results; needs verification
	NCT03386877	Autologous DPSCs	Randomized, scaffold-assisted	—	Completed	CAL gain, radiographic bone fill	No results posted; European pilot
Oral Submucous Fibrosis	NCT04608838	DPSCs	Interventional, open-label	42 (actual)	Completed	Mouth opening, mucosal elasticity	Completed; publications listed but no registry results
Huntington’s Disease	NCT02728115	Allogeneic DPSCs	Phase I, safety	6	Completed	Safety, neurological assessment	No results posted; very small early trial
Dental Pulp Regeneration	NCT01814436	Autologous SHEDs	Single-arm	80 (planned)	Unknown (last update 2016)	Root development, apical closure	No results posted; high risk of attrition
	NCT00595595	DPSCs	Early interventional	—	Historic, uncertain	Pulp vitality, root development	No registry results; very early study
Alveolar Cleft Repair	NCT01932164	DPSCs	Interventional	—	Completed (results posted)	Alveolar bone fill	Results posted; supportive evidence
	NCT03766217	Allogeneic DPSCs + biomaterials	Phase III, randomized vs. iliac crest bone graft	62 (actual)	Completed (2019)	Bone fill, canine eruption	Completed; no registry results, publications listed

Meanwhile, there is increasing evidence that DSCs have functional differences in osteogenic differentiation, proliferation rate and angiogenic potential (Okić-Đorđević et al., 2021). Therefore, for DSCs we need to further explore the function of different types of DSCs and perform tissue-specific labeling. Based on this, there are no definitive studies on how to determine the standard process of DSCs therapy, how to clarify the optimal transplantation time, quantity, and form of injection, as well as whether there is a postoperative response and long-term efficacy, which all need to be further explored. Advances in single-cell lineage tracing technology hold great promise for elucidating the heterogeneity and specific functions among different types of DSCs and may hold the key to solving this problem.

To sum up, future progress will require international collaboration to harmonize research and clinical practices. Establishing unified Good Manufacturing Practice (GMP) protocols for MSCs processing and quality control, as well as building an international stem cell consortium or alliance, could provide shared resources, standardize endpoints, and facilitate large-scale multicenter trials. Moreover, integrating omics technologies to clarify mechanisms and advancing acellular strategies such as exosome- or secretome-based therapies may help overcome current barriers. By addressing these challenges, MSCs-based therapies—including DSCs and other sources—may ultimately evolve into reliable, safe, and personalized approaches for regenerative medicine. But that is still a long way to go.

## Glossary

DSCs: Dental Stem Cells
DPSCs: Dental Pulp Stem Cells
PDLSCs: Periodontal Ligament stem Cells
SHEDs: Stem Cells from Human Exfoliated Deciduous Teeth
DFSCs: Dental Follicle Stem Cells
BMSCs: Bone Marrow Stem Cells
UCMSCs: Umbilical Cord Mesenchymal Stem Cells
ADSCs: Adipose-derived Stem Cells
MSCs: Mesenchymal stem cells
MHC-I: Major Histocompatibility Complex Class I
MHC-II: Major Histocompatibility Complex Class II
CDE: Center for Drug Evaluation
BMP-2: Bone Morphogenic Protein-2
GMSCs: Gingiva-derived Mesenchymal Stem Cells
SCAPs: Stem Cells from Apical Papilla
iSCAPs: Immortalized Stem Cells from Apical Papilla Lines
ALP: Alkaline Phosphatase
NSE: Neuron-specific Enolase
VEGF-B: Vascular Endothelial Frowth Factor-B
GDNF: Glial Cell Line-derived Neurotrophic Factor
iPSCs: Induced pluripotent stem cells
PCNA: Proliferating Cell Nuclear Antigen
FGF: Fibroblast Growth Factor
PDT: Population Doubling Time
PGE-2: Prostaglandin E2
IDO: Indoleamine-2,3-dioxygenase
iNOS: Inducible Nitric Oxide Synthase
TGF-β: Transforming Growth Factor-beta
COX-2: Cyclooxygenase-2
SHEDs-CM: Stem Cells from Human Exfoliated Deciduous Teeth Conditioned Medium
ED: Siglec-9-Ectodomain of Sialic Acid-binding Ig-like Lectin-9
HGF: Hepatocyte Growth Factor
Treg: Regulatory T Cells
EVs: Extracellular Vesicles
DPSCs-Exo: exosomes derived from DPSCs
TNF-α: Tumor Necrosis Factor-alpha
ADO: Adenosine
ATP: Adenosine Triphosphate
CHS: contact hypersensitivity
DC: Dendritic Cell
MC: Mast Cell
G-CSF: Granulocyte Colony-Stimulating Factor
RA: Rheumatoid Arthritis
SLE: Systemic Lupus Erythematosus
UCHL1: Ubiquitin C-terminal hydrolase L1
TUBB3: Class III b Microtubulin
MAP2: Microtubule-associated Protein 2
BDNF: Brain-derived Neurotrophic Factor
NGF: Nerve Growth Factor
NT-3: Neurotrophin-3
DACC: Dental Apical Complex Cells
NCCs: Neural Crest Cells
IBD: Inflammatory Bowel Disease
AMSCs: Amniotic Membrane Mesenchymal Stem Cells
NSCs: Neural Stem Cells
I/R: Ischemia-Reperfusion
RGCs: Retinal Ganglion Cells
rhIGFBP5: Recombinant Human IGFBP5 Protein
IL: 1RA-Interleukin-1 Receptor Antagonist
DMP-1: Dentin Matrix Protein-1
OM: Odontogenic Medium
GelMA: Gelatin Methacryloyl
DSPP: Dentin Sialophosphoprotein
GMP: Good Manufacturing Practice
CAL: clinical attachment level
VAS: visual analogue scale
WOMAC: Western Ontario and McMaster Universities Osteoarthritis Index
